# Eliazar de Wind (1916–1987)

**DOI:** 10.1007/s00415-020-09922-0

**Published:** 2020-05-23

**Authors:** Jarosław Sak, Magdalena Suchodolska

**Affiliations:** 1 Department of History and Philosophy, Medical University of Lublin, ul. Chodźki 7, 20-093 Lublin, Poland; 2 Faculty of Medicine, Medical University of Lublin, Lublin, Poland

**Fig. 1:**
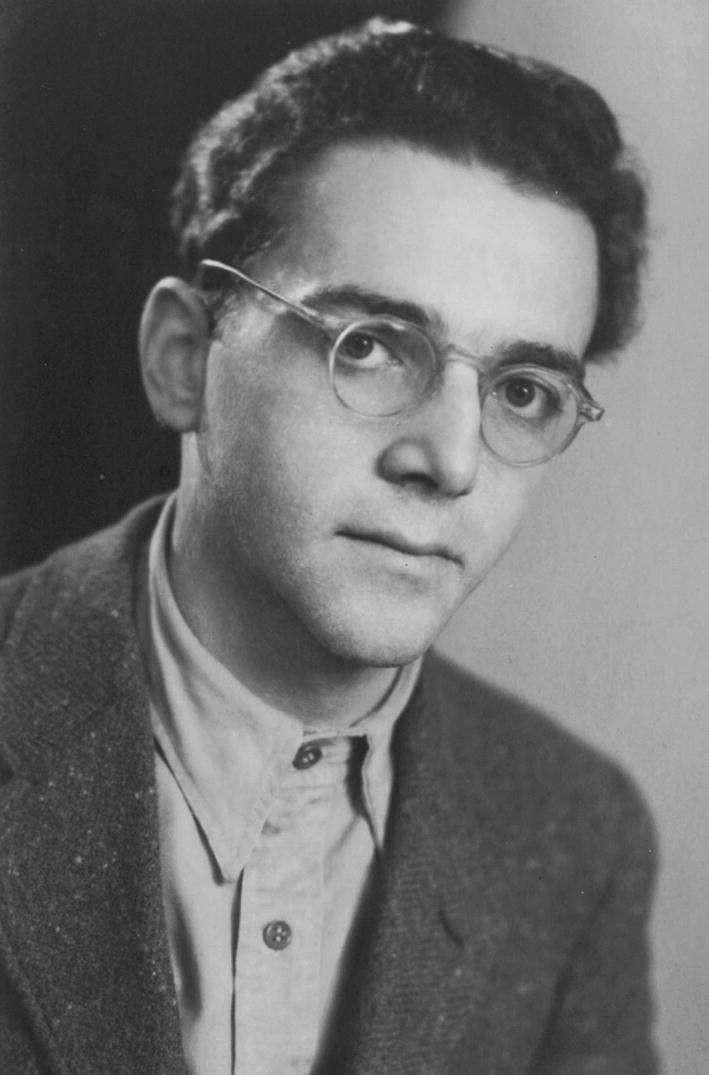
Eliazar de Wind (1916–1987). The photo from https://www.hachettebookgroup.com/contributor/eliazar-de-wind/

2020 marks the 75th anniversary of the liberation of the German Nazi concentration and extermination camp, Auschwitz. It was the main place of the Holocaust of European Jews during World War II. One of the few Auschwitz prisoners who survived the Holocaust was Eliazar (Eddy) de Wind (1916–1987), a Dutch doctor—psychiatrist and psychoanalyst of Jewish descent (Fig. 1). He was the first who recognized the concentration camp syndrome (KZ syndrome) [1–3]. This syndrome became the precursor of “Post Traumatic Stress Disorder” (PTSD) in the new Diagnostic and Statistical Manual III (DSM III) [4]. He also drew attention to the transgenerational transmission of camp trauma [3, 5, 6].

Eliazar (Eddy) de Wind was born in The Hague on February 6, 1916 as the only child in a Jewish family. His parents were Louis de Wind (1892–1919) and Henriëtte Sanders (1892–1942). He was brought up by his mother as his father died of brain cancer when Eddy was three years old. Eddy’s childhood and adolescence fell in the years preceding World War II. His great passion was sailing on the lakes and canals near The Hague [3]. After graduating from high school, he started medical studies at Leiden University—the oldest Dutch university. He was a talented saxophonist and clarinettist and played in the student jazz band The Rhythm Rascals. He completed his medical studies in 1940 as the last of the Jewish students who graduated from Leiden University in the early period of the German occupation [3].

In November 1940, the German authorities ordered the lay-off of all Jewish university employees and the removal of Jewish students. This led to protests by university communities throughout the Netherlands. Dean and Professor of law at Leiden University, Rudolph Pabus Cleveringa (1894–1980) protested against the dismissal of Jewish colleagues ordered by the German occupation authorities in his speech of 26 November, 1940. After the closure of Leiden University by the Nazi occupation authorities, there was a break in its functioning until 1945. After graduating, Eddy took part in secret courses run by lecturers in private homes in Amsterdam to get a specialization in psychoanalysis. In 1942, he returned to his home in The Hague.

In 1942, the authorities of the Third Reich implemented a plan to exterminate eleven million European Jews living in the occupied territories. From among the 140,000 Jewish people living in the Netherlands, only 27 percent survived the German occupation [7]. Dutch Jews were imprisoned in the transit camp in Westerbork prior to transportation to extermination camps in Eastern Europe, mainly to Auschwitz and Sobibor. The first deportation from the Westerbork camp to Auschwitz took place on July 15, 1942 [7, 8].

Eddy volunteered to work as a doctor in Westerbork because his mother was imprisoned there. In May 1943, in this camp, Eddy met and married a nurse—Friedel Komornik. On September 14, 1944, he and his wife were sent to Auschwitz [3]. Despite the dramatic existential conditions, they managed to survive. Before the evacuation of the Auschwitz prisoners to concentration camps in Germany and Austria, he was hiding to avoid the death march. His wife did not manage to do so; and along with other prisoners was led by SS soldiers into Germany. On January 27, 1945, the extermination camp Auschwitz was liberated by the Soviet Army. Eddy stayed in the camp—already as a free man—for several months after its liberation, treating former prisoners. He performed small surgical procedures at that time [3]. During these several months he wrote down memories of the imprisonment in Westerbork and Auschwitz.

In the summer of 1945, he returned to the Netherlands and found his wife Friedel, saved from the death march. He tried to start a new life with his wife, but did not forget about the recent traumatic experiences. He built a house on the outskirts of Amsterdam, where they started a new life. In 1946, Eddy published his Dutch camp memories in the book “Eindstation Auschwitz. Mijn verhaal vanuit het kamp (1943–1945)” (“Last Stop Auschwitz. My story from the camp (1943–1945)” [3]. He worked as a psychiatrist and psychoanalyst specializing in the treatment of severe war trauma. He founded the Foundation for Research on the Psychological Effects of War (Stichting Onderzoek Psychische Oorlogsgevolgen—SOPO). Eddy was awarded the Order of Orange-Nassau by Queen Regnant of the Netherlands in 1984. He died on September 27, 1987.

Eddy de Wind was the first who presented a clinical description of the concentration camp syndrome (KZ syndrome) in an article “Confrontatie met de dood” (“The confrontation with death”) published first in Dutch in 1949 [1, 2] He analysed psychological mechanisms of defence and adaptation of prisoners and described stages of the formation of camp trauma. Eddy pointed to the connection between social structure of the camp and the changes in prisoners’ psyche [1–3]. In his further studies he pointed out groups of KZ symptoms: recurrent, unwanted distressing memories of the traumatic event, social withdrawal and hyperarousal. He noted the deep acceptance and conviction of the inevitability of death that occurred in the KZ syndrome. He emphasized that some of the symptoms may be passed on by parents to their children [3, 5, 6]. This applies especially to difficulties in maintaining close relationships and a sense of constant danger. This phenomenon of transgenerational transmission of trauma has an impact on the personality development of the second generation, i.e. the children of former prisoners [5, 6].

Research on the KZ syndrome initiated by Eddy created a clinical picture of psychological and neurological disorders caused by traumatic experiences [1, 2, 5, 9], as well as leading to therapy [10]. It is worth emphasizing that currently this kind of disorder belongs to PTSD, which concept was restored to DSM-III in 1980, also thanks to the work of William G. Niederland (1904–1993) [4]. The illness effects of the post-camp syndrome still affect living former prisoners of Nazi concentration camps and their children.
